# D-Lactic Acidosis in Short Bowel Syndrome

**DOI:** 10.7759/cureus.25471

**Published:** 2022-05-30

**Authors:** Ayham Khrais, Hasan Ali, Sung Choi, Ahmed Ahmed, Sushil Ahlawat

**Affiliations:** 1 Internal Medicine, Rutgers University New Jersey Medical School, Newark, USA; 2 Pulmonary and Critical Care, Rutgers University New Jersey Medical School, Newark, USA; 3 Gastroenterology and Hepatology, Rutgers University New Jersey Medical School, Newark, USA; 4 Gastroenterology and Hepatology, Rutgers University, Newark, USA

**Keywords:** anion gap mmetabolic acidosis, d-lactic acidosis, crohn’s disease (cd), lactic acidosis, short bowel syndrome

## Abstract

D-lactic acidosis (D-LA) is closely associated with short bowel syndrome (SBS). Decreased intestinal absorption results in the delivery of carbohydrates to the colon, where the fermentation by colonic flora leads to D-LA. Systemic absorption of D-lactic acid results in anion-gap metabolic acidosis (AGMA), LA, and neurologic symptoms. In this report, we describe the case of a 43-year-old man with Crohn’s disease (CD) and bowel resection who presented with abdominal pain and slurred speech. He was found to have AGMA and persistent LA despite receiving intravenous fluids, which improved after carbohydrate restriction. A high index of suspicion for D-LA should be maintained when encountering patients who have undergone bowel resection and with unexplained AGMA.

## Introduction

Lactic acidosis (LA) in a patient presenting with abdominal pain raises concern for a wide differential, including bowel ischemia, colitis, or appendicitis [1]. The physician is often prompted to administer intravenous fluids, locate the involved area, and consider medical management and possible invasive procedures. However, different isomers of lactic acid exist, with the standard lactic acid laboratory value being unable to distinguish between these isomers [1,2]. The elevation of D-lactic acid may signify an alternative diagnosis; however, levels of D-lactic acid are not routinely measured in patients with elevated lactic acid, and they cannot be measured in a timely manner [1]. Therefore, a high clinical suspicion is required for the diagnosis and management of D-lactic acidosis (D-LA). Having a proper understanding of D-LA can guide physicians toward providing effective treatment and preventing the harm caused by unnecessary imaging and invasive interventions.

## Case presentation

A 43-year-old man with Crohn’s disease (CD) complicated by bowel perforation status post bowel resection with ileostomy and reversal, complicated by multiple abdominal wall hernias status post repairs, presented with two weeks of diffuse abdominal pain, as well as four days of hematemesis and hematochezia. Of note, he had not been prescribed any medications for CD. On admission, he was hypertensive (155/101 mmHg), tachycardic (118 beats per minute), and somnolent with slurred speech. His abdomen was soft but diffusely tender with guarding but no rebound tenderness, with scarring from previous abdominal surgeries. The rectal exam was notable for external hemorrhoids and perianal disease, without frank blood or melena. Laboratory evaluation revealed a white blood cell count of 29 and an anion-gap metabolic acidosis (AGMA) with an anion gap (AG) of 20, bicarbonate of 24, and lactic acid level of 4.3 (Tables 1, 2). CT scan of the abdomen and pelvis without contrast was unremarkable. The patient was admitted for the management of AGMA in the setting of elevated serum lactic acid and possible CD flare.

**Table 1:** Complete blood count and metabolic panel measured during the initial presentation

Variable	Patient’s value	Reference range	Units
White blood cell (WBC) count	29	4.5–11.0	x 10^9^ cells/L
Red blood cell (RBC) count	4.3	4.3–5.9 (male), 3.5–5.5 (female)	x 10^12^ cells/L
Hemoglobin	11.6	2.09–2.71 (male), 1.86–2.48 (female)	g/dL
Hematocrit	35.8	0.41–0.53 (male), 0.36–0.46 (female)	%
Platelets	264 x 10^9^	150–400	x 10^9^/L
Glucose	280	64–100	mg/dL
Sodium	144	136–144	mEq/L
Potassium	3.8	3.7–5.2	mEq/L
Chloride	104	96–106	mEq/L
Carbon dioxide (CO_2_)	24	23–29	mEq/L
Blood urea nitrogen (BUN)	9	6–20	mg/dL
Creatinine	0.7	0.8–1.2	mg/dL
Calcium	9.7	8.5–10.2	mg/dL
Magnesium	2.0	1.7–2.2	mg/dL
Phosphorus	1.6	3.0–4.5	mg/dL

**Table 2:** Fluctuating serum lactic acid levels in chronological order from left to right

Hospital day	1	1	1	2	2	2	3	3	4	5
Lactic acid levels (mmol/L)	4.3	5.9	5.4	2.5	1.6	6.7	2.7	4.3	2.5	1.8

**Figure 1:**
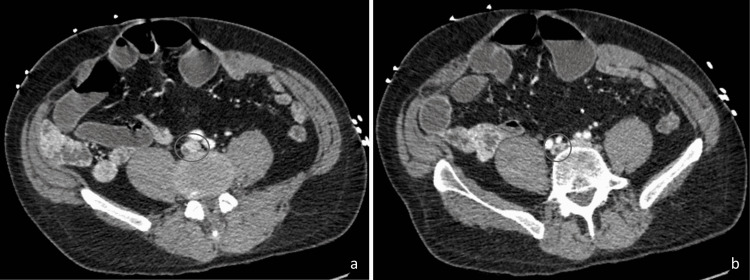
CT scan of the abdomen and pelvis with and without intravenous contrast demonstrating non-occlusive thrombi (circle) within the IVC (a) and the right common iliac vein (b) CT: computed tomography; IVC: inferior vena cava

In line with the workup for AGMA, testing for ketoacidosis and toxic ingestion was performed; however, the results were negative. Gastrointestinal pathogen panel and *Clostridium difficile* testing performed to rule out infectious causes of diarrhea in light of the patient’s self-reported history of *Clostridium difficile* infection (CDI) were negative as well. Lactic acid remained elevated despite the infusion of intravenous fluids. The patient was therefore evaluated for possible bowel ischemia as the etiology of the persistent LA. CT scan of the abdomen and pelvis with and without intravenous contrast showed two non-occlusive thrombi, one within the right common iliac vein and one in the inferior vena cava (Figure 1). CT showed no fat stranding, bowel wall thickening, or enhancement to suggest CD flare. Given the presence of multiple thrombi and laboratory signs of bowel ischemia in the setting of normotension and hypertension, a diagnosis of mesenteric ischemia was assumed and therapeutic heparin was started. The patient was maintained on meropenem for the assumed diagnosis of ischemic bowel. Vascular surgery was consulted, and they recommended an inferior vena cava (IVC) filter given the right iliac vein thrombosis; however, the patient refused to undergo this procedure.

Gastroenterology was consulted given the patient’s initial presentation of hematochezia and melena in the setting of his known history of CD, and the patient underwent esophagogastroduodenoscopy (EGD) and colonoscopy. EGD was significant for two clean-based antral ulcers and gastritis, without active bleeding or signs of infection in the esophagus or duodenum. Colonoscopy showed internal and external hemorrhoids, indicating prior colectomy, anal condyloma, and proctitis, but no evidence of ileal disease. The patient was maintained on intravenous pantoprazole twice a day afterward.

Hemoglobin remained stable and the patient exhibited no episodes of hematochezia or melena during his hospital stay. He was observed to be consuming juice and several packets of sugar with tea, raising concerns for D-LA. After consultation with the chemistry laboratory department, we confirmed that D-lactic acid and L-lactic acid are not specifically identified in the lactic acid laboratory value, and hence D-lactic acid level was sent out to test for this isomer specifically. After placing the patient on a diabetic diet, restricting carbohydrates, and eliminating sugar packets, we noted that lactic acid levels decreased to 1.8 (Table 2) and the AGMA normalized with a bicarbonate of 23 and AG of 12. The blood sample sent out previously showed an elevated D-lactic acid of 0.5 mM (reference level: <0.2 mM).

## Discussion

This patient’s neurologic symptoms, AGMA and LA, simple carbohydrate intake, and prior bowel resection raised concerns for D-LA, a rare condition observed in patients with short bowel syndrome (SBS). SBS denotes malabsorption secondary to a significant loss of bowel length. Malabsorption results from fluid and electrolyte derangements due to decreased absorptive surface area and bowel motility [1-3]. Therefore, a significant amount of unabsorbed, partially digested nutrients passes downstream to the colon, which is responsible for fluid and fatty acid absorption, mediated in part by colonic flora [4-6]. Gut bacteria convert partially digested carbohydrates into short-chain fatty acids (SCFA). This reaction also produces small concentrations of lactic acid as a byproduct, due to the presence of D- and L-lactate dehydrogenase in some bacterial species. Levels of both lactic acid isomers are normally low, as lactate is eliminated via conversion into SCFA at the same rate at which it is produced.

D-lactate overproduction is caused by the presence of a shortened small intestine (SI), and the overgrowth of bacteria containing D-lactate dehydrogenase. A decrease in SI length, as seen in SBS, results in carbohydrate malabsorption, causing them to persist distally to the colon, where they become available as chemical substrates for bacteria [5]. Within the colon, gut flora ferment carbohydrates, resulting in the overproduction of SCFA and lactate. Accumulation of these organic acids increases the acidity of the colon, promoting the growth of acid-resistant bacteria (e.g., Lactobacilli) that contain higher levels of D- and L-lactate dehydrogenase [5]. This alteration in gut microbiota results in the excess production of D- and L-lactate in the gut, followed by absorption into the bloodstream.

Systemic absorption can cause AGMA, LA, and neurologic symptoms, such as encephalopathy, dysarthria, and ataxia. While patients with D-LA have neurologic findings, D-lactate has no role in their presentation [4,5]. Instead, it is hypothesized that encephalopathy in D-LA is due to the excess production of other organic acids (e.g., aldehydes, alcohols) in conjunction with D-lactate, which, upon absorption, can result in neuronal toxicity. Neurotoxicity can be observed in the cerebellum, manifesting as clumsiness, ataxia, and unsteady gait. It can also result in decreased concentration, hallucinations, and weakness [4].

The diagnosis of the condition is based on clinical suspicion, AGMA that resolves with carbohydrate restriction, and neurologic symptoms. D-lactate dehydrogenase assay confirms the diagnosis. In patients with AGMA, standard serum lactate is often obtained; however, it contains only L-lactate dehydrogenase, and hence cannot distinguish the levels of serum D-lactate from L-lactate. Specialized assays containing D-lactate dehydrogenase must be used in order to quantify serum D-lactate concentrations [7]. Unfortunately, these assays are usually sent to outside laboratories and it often takes days to obtain the results. Alternatively, stool D- and L-lactate levels can be measured. A strong correlation between the ratio of fecal D- and L-lactate (D/L ratio) and the risk of encephalopathy in patients with D-LA has been documented. Patients with D/L ratios <1 remained asymptomatic while those with D/L ratios >2 were at higher risk for encephalopathy [7]. Therefore, fecal D- and L-lactate levels can be used to diagnose and stratify patients with D-LA based on their risk of becoming symptomatic.

In contrast to L-LA, serum AG in D-LA is lower than the decrease in serum bicarbonate, and can thus be normal. Therefore, D-LA may also result in hyperchloremic metabolic acidosis [1,5,8]. This is due to the inefficient reabsorption of D-lactate by the kidney, caused by the lack of D-lactate-specific receptors in the lumen of the proximal tubule. D-lactate is excreted with cations such as sodium, and hence excess urinary excretion of D-lactate results in a decrease in extracellular fluid, thereby causing increased renal retention of sodium chloride. This ultimately results in hyperchloremic metabolic acidosis. As such, D-LA should still be suspected in patients with SBS, encephalopathy, and a normal AGMA.

The treatment involves the correction of acidosis and oral carbohydrate restriction. A low-carbohydrate diet will limit the amount of substrate available to the D-lactic dehydrogenase enzyme found in enteric bacteria, effectively reducing the production of D-lactic acid [5]. Enteric antibiotic use is proposed to reduce acid-forming bacteria, aiding in symptom control and disease resolution [3]. Examples include vancomycin, metronidazole, and ampicillin [2-4,9].

Physicians should suspect D-LA in patients with CD post-bowel resection and otherwise unexplained AGMA, as this could prevent harm caused by inappropriate treatment including intravenous antibiotics and corticosteroids, radiation exposure, and invasive endoscopic evaluation.

## Conclusions

D-LA should be suspected in patients with SBS who present with AGMA with elevated serum lactic acid and otherwise unremarkable workup for other etiologies of the aforementioned laboratory values. Elevated serum D-lactic acid is prevalent in SBS due to carbohydrate delivery to the colon, allowing fermentation by microorganisms. Therefore, in patients with suspected D-LA, carbohydrate restriction should be preemptively started with subsequent monitoring for improvement in symptoms and laboratory values.

## References

[1] Bakhru MR, Kumar A, Aneja A (2007). A 58-year-old woman with mental status changes. Cleve Clin J Med.

[2] Bianchetti DG, Amelio GS, Lava SA (2018). D-lactic acidosis in humans: systematic literature review. Pediatr Nephrol.

[3] James PD, Black D, Kuper A, Saibil F (2010). D-lactic acidosis and ataxia in a man with Crohn disease. CMAJ.

[4] Kang KP, Lee S, Kang SK (2006). D-lactic acidosis in humans: review of update. Electrolyte Blood Press.

[5] Kowlgi NG, Chhabra L (2015). D-lactic acidosis: an underrecognized complication of short bowel syndrome. Gastroenterol Res Pract.

[6] Limketkai BN, Parian AM, Shah ND, Colombel JF (2016). Short bowel syndrome and intestinal failure in Crohn's disease. Inflamm Bowel Dis.

[7] Mayeur C, Gratadoux JJ, Bridonneau C (2013). Faecal D/L lactate ratio is a metabolic signature of microbiota imbalance in patients with short bowel syndrome. PLoS One.

[8] Stanciu S, De Silva A (2018). Metabolic acidosis in short bowel syndrome: think D-lactic acid acidosis. BMJ Case Rep.

[9] Uribarri J, Oh MS, Carroll HJ (1998). D-lactic acidosis. A review of clinical presentation, biochemical features, and pathophysiologic mechanisms. Medicine (Baltimore).

